# Ursodeoxycholic acid use in lactating female patients is associated with clinically negligible concentrations of this bile acid in breast milk

**DOI:** 10.1038/s41598-022-24253-y

**Published:** 2022-11-15

**Authors:** Patrik Šimják, Tomáš Petr, Barbora Kaslová, Tomáš Fejfar, Petr Hůlek, Antonín Pařízek, Libor Vítek

**Affiliations:** 1 Department of Gynecology and Obstetrics, General University Hospital in Prague and 1st Faculty of Medicine, Prague, Czech Republic; 2 Institute of Medical Biochemistry and Laboratory Diagnostics, General University Hospital in Prague and 1st Faculty of Medicine, Prague, Czech Republic; 3 Department of Internal Medicine, University Hospital in Hradec Králové and Faculty of Medicine in Hradec Králové, Hradec Králové, Czech Republic; 4 4th Department of Internal Medicine, General University Hospital in Prague and 1st Faculty of Medicine, Charles University in Prague, U Nemocnice 2, Prague 2, 128 08 Czech Republic

**Keywords:** Biochemistry, Gastroenterology

## Abstract

In the literature on the safety of ursodeoxycholic acid (UDCA) during breastfeeding, insufficient data has been reported to date. Thus, the aim of our study was to analyze bile acid (BA) concentrations in breast milk in a cohort of patients, treated with UDCA, and with various cholestatic liver diseases. The study was carried out on a cohort of 20 patients with various cholestatic diseases. All the patients were treated with UDCA (500–1500 mg daily). Concentrations of BA, sampled on day 3 after delivery were analyzed using the GS-MS technique, and then compared to untreated women. Total BA concentrations in the breast milk of the UDCA-treated patients were equal to those of the untreated women controls (3.2 ± 1 *vs.* 3.2 ± 0.2 µmol/L, respectively). The UDCA concentrations in breast milk remained negligible in UDCA-treated patients (0.69 µmol/L), and in any event did not contribute to the newborn BA pool. No apparent side-effects of the maternal UDCA treatment were observed in any newborn infant, and no deterioration in postnatal development was observed during the routine 1-year follow-ups. Therapeutic administration of UDCA during lactation is safe for breastfed babies since UDCA only gets into breast milk in negligible amounts. UDCA treatment should be allowed and included into the guidelines for the therapy of cholestatic diseases in breastfeeding mothers.

## Introduction

Ursodeoxycholic acid (UDCA) is currently used as treatment of choice in patients with a variety of cholestatic liver diseases^1–3^. Although UDCA is not officially approved by the drug administration agencies for use during pregnancy, it can be administered to patients with cholestatic liver diseases with no harm to either the mothers or fetuses^1^. UDCA is also not approved during breastfeeding; however, based on the scarce data thus far published, this treatment is believed to be safe for newborns^1,3–5^. Given the generic availability of UDCA and accepted safety in pregnant women, there is minimal effort on a larger scale to generate and perform new clinical research studies using this drug. Despite its common use in pregnancy, the data on UDCA concentrations in the breast milk of treated patients remains insufficient. In fact, there are only a few reports in the literature concerned with this topic, reporting either none^6^ or only negligible appearances of UDCA in the breast milk of treated patients^7^. We have also previously published a case report of a breastfeeding patient with primary biliary cholangitis (PBC) treated with increasing doses of UDCA, with careful monitoring of bile acids (BA) in her serum and breast milk. This case study proved that UDCA did not transfer into the breast milk in this particular patient in any clinically relevant amount^8^.

Based on that case report, we initiated a larger study to confirm this data of low concentrations of UDCA in breast milk on larger population to find out whether or not an inter-individual variability exists in breast milk BA concentrations, and to establish a basis for reconsideration of the safety of UDCA treatment during breast-feeding by the drug administration authorities as well as UDCA manufacturers.

## Materials and methods

### Patients

The study was carried out on a cohort of 20 patients (aged 23–38 years) with various cholestatic diseases, in whom there was a clear indication to continue UDCA (patients with PBC, n = 4) or it was appropriate to continue this treatment (patients with intrahepatic cholestasis of pregnancy (ICP), (n = 15), and hemolysis, elevated liver enzymes, and low platelet count (HELLP) syndrome (n = 1)). All of the patients were treated with UDCA (500–1500 mg daily) until the 3rd day after delivery. The dose of UDCA was determined according to the guidelines of the Czech Society of Gynecology and Obstetrics for treatment of ICP (500–1500 mg/day)^9^ and EASL guidelines for treatment of PBC (13–15 mg/kg/day)^3^. A blood serum and breast milk samples were collected on day 3 after delivery for further analysis of BA. Patients with PBC continued with UDCA therapy, whereas the remaining patients (mainly ICP patients) stopped treatment, since it was no longer required. One patient manifested with PBC 3 weeks after delivery, and her breast milk sample was collected 4 weeks after delivery, as previously reported^8^. The data from this patient was also included into the current cohort analysis.

Four lactating women were used as controls, to compare the BA concentrations in human milk (sampled again on the 3rd day after delivery). All the subjects were on isocaloric, balanced diet; there were no additional health issues apart from cholestatic liver disease in the study group.

Standard clinical examination of infants during early postnatal period and routine pediatric examinations during 1st year of life were performed in all infants.

Written informed consent was obtained from each patient included in the study, and the study protocol conformed to the ethical guidelines of the 1975 Declaration of Helsinki as reflected in a priori approval by the Ethics Committee of the General University Hospital in Prague.

### BA determination

Serum BA concentrations (cholic acid (CA); chenodeoxycholic acid (CDCA); deoxycholic acid (DCA); lithocholic acid (LCA) and UDCA) were measured by gas chromatography/mass spectroscopy (GC/MS; Agilent 5973 Network Mass Selective Detector, 6890N Network GC System, Santa Clara, CA, USA) as described previously^10,11^. In brief, BA along with an internal standard (23,24-bisnor-3βOH 5α cholic acid, Steraloids, Inc., Newport, RI, USA) were extracted from serum samples using SXG C18 columns, (Tessek, Czech Republic); their conjugates were hydrolyzed by 10% KOH and acidified by 10 M HCl. The BA were washed on SXG C18 columns and then converted onto methylsilyl derivatives. All GC analyses were performed on a capillary column (5% phenylmethylpolysiloxane, HP5MS, 15 m × 0.25 mm ID, 0.25 mm, ValcoBond, VICI Valco Instruments, Houston, TX, USA) with MS detection (quadruple mass spectrometer operating in negative mode). The sensitivity of this method was 0.04 mmol/L, and the reproducibility was 6.4%.

### Statistical analyses

The data are expressed as the mean ± SD, or as the median and IQ range when the data were non-normally distributed. The Mann–Whitney rank sum test was used to compare BA concentrations in the breast milk. All analyses were performed with the alpha set to 0.05. The statistics were computed using SigmaPlot v. 14.5 (Systat Software, Inc., San Jose, CA, USA).

## Results

Serum and breast milk BA concentrations were analyzed in all patients treated with UDCA. Their total serum concentrations were relatively low (32.7 ± 23 µmol/L) with contribution of UDCA of more than 50%, indicating potent choleretic effects of UDCA and substantial enrichment of BA pool with UDCA (Table 1). Interestingly, total BA concentration in breast milk did not differ between the UDCA-treated and non-treated females, being rather low and completely equal (3.2 ± 1 *vs.* 3.2 ± 0.2 µmol/L, respectively, Table 1). As expected, UDCA proportion in breast milk of treated patients was much higher compared to untreated women (21.5 *vs*. 1.5%, p = 0.003), and a trend to much higher proportion of lithocholic acid (LCA) in breast milk of UDCA-treated patients was also clearly found (Table 1). Increased production of LCA was most likely a result of the conversion of UDCA on LCA^12,13^, catalyzed by maternal gut anaerobic microbiota possessing BA 7β-dehydroxylase activity^14^.

**Table 1:** BA concentrations in sera and human milk of breast-feeding women treated with UDCA.

UDCA-treated patients (n = 20)											
Serum BA [µmol/L]	CA [%]	CDCA [%]	DCA [%]	LCA [%]	UDCA [%]	Milk BA [µmol/L]	CA [%]	CDCA [%]	DCA [%]	LCA [%]	UDCA [%]
**32.7** ± 23 **24** (12–42)	**20.3** ± 14 **16** (8–29)	**16.7** ± 8 **14** (11–20)	**7.7** ± 2.6 **9** (5–9)	**4.1** ± 4 **2** (2–5)	**52.2** ± 22 **60** (39–67)	**3.2** ± 1	**30.5** (22–37)	**24.5** (20–30)	**13** (10–19)	**7.5** (2.3–11)	**21.5** (17–26)
**Untreated controls (n = 4)**											
Serum BA	ND	ND	ND	ND	ND	**3.2** ± 0.2	**34** (28–51)	**37** (34–45)	**21** (7–22)	**1** (1–13)	**1.5** (1–3.5)
*P*-value						0.668	0.333	**0.006**	0.245	0.183	**0.003**

Data presented as mean ± SD and/or median and interquartile range.

BA, bile acids; CA, cholic acid; CDCA, chenodeoxycholic acid; DCA, deoxycholic acid; LCA, lithocholic acid; UDCA, ursodeoxycholic acid; ND, not done.

Significant values are in bold.

When comparing the total load of UDCA in neonates exposed to the breast milk of UDCA-treated and non-treated women, and assuming a maximal daily intake of breast milk to be 100 mL^15^, the daily intake of UDCA from breast milk would only be 27 mg (Table 2). Taking into account the total BA pool in full-term newborn infants, which is approximately 60 mg^16^, a daily intake of 27 µg of UDCA would only represent 0.0005% of the total newborn BA pool.

**Table 2:** Estimate of the BA load in the presumed daily intake of human milk (100 mL) of breast-feeding women treated with UDCA.

	Total BA [µg]	CA [µg]	CDCA [µg]	DCA [µg]	LCA [µg]	UDCA [µg]
UDCA-treated	126	40	31	16	3	27
Controls	126	44	46	26	0.4	1.9

As a daily dose, 100 mL of human milk was used to assess the newborn´s load of ingested BA^16^.

BA, bile acids; CA, cholic acid; CDCA, chenodeoxycholic acid; DCA, deoxycholic acid; LCA, lithocholic acid; UDCA, ursodeoxycholic acid.

The same negligible impact was estimated for LCA exposure as well as deoxycholic acid (DCA) plus exposure to LCA (DCA and LCA representing hydrophobic, and potentially more toxic BA^17^) (Table 2).

No apparent side-effects of the maternal UDCA treatment were observed in any newborn infant based on standard clinical examination during early postnatal period, and no deterioration in postnatal development was observed during routine 1-year follow-up on routine pediatric examinations.

## Discussion

There is reluctance among general practitioners, internal medicine but also hepatologists to use UDCA for the treatment of breastfeeding patients who suffer from cholestatic diseases, precisely because this drug is not officially approved for use during lactation by the drug authorities.

However, several lines of evidence suggest that this therapy might be safe for breastfed babies, as in a previous study no UDCA was detected by HPLC in the milk of a woman breastfeeding, having been treated with UDCA at a dose of 750 mg/day (most likely due to sensitivity limits of the method used to detect submicromolar concentrations of UDCA)^6^. This was further reinforced in a larger study by Brites and Rodrigues, in which BA concentrations were studied in the colostrum of seven lactating patients with ICP, treated with UDCA at a dose of 14 mg/kg/body weight daily until delivery. The concentrations of UDCA in colostrum was found to be very low, reaching 0.3 ± 0.2 mmol/L in treated patients; and that the breastfeeding infants were estimated to be exposed to as low a total daily dose of UDCA as approximately 12 mg^7^. Even more importantly, UDCA was undetectable in human milk samples collected 12 h after administration of 500 mg of UDCA to selected patients^7^.

In concordance with this data, no adverse reactions were reported in a breast-fed infant of a mother with advanced PBC, daily treated with 750 mg of UDCA^18^. Similar results were also reported in a case report by Erol-Coskun in a breast-feeding mother treated with 500 mg of UDCA for cholestasis accompanying hypothyroidism^15^. A retrospective Turkish study on breastfeeding patients with PBC treated with UDCA, also consistently revealed no adverse reactions in breastfed infants^19^.

Speaking of the safety of UDCA therapy during lactation, the second line of evidence comes from clinical neonatal studies on a variety of neonatal conditions treated with UDCA, in which this treatment is generally well tolerated, and free of either immediate side effects or long-term unfavorable sequels.

These diseases affecting newborns that prominently included neonatal cholestatic conditions as well as neonatal jaundice; and noting that the newborns responded well to UDCA treatment. Because of its choleretic effect, UDCA (10–30 mg/kg/day) has generally been used in patients with neonatal cholestasis of various etiologies, including biliary atresia patients who had undergone portoenterostomy with no serious health concerns reported^20,21^. The safety of UDCA treatment (doses up to 30 mg/kg/day) was also proven in preterm, very low birth weight neonates, including those suffering from parenteral nutrition-associated cholestasis^22–29^. It is also important to note that prenatal UDCA exposure in mothers treated with UDCA for ICP did not reveal any adverse signs by the 12th month after delivery infant follow-up^30^. UDCA treatment in Niemann-Pick type C-related cholestatic disease was also found safe in patients treated early in the postnatal period^31^.

Finally, as we reported in our previous experimental studies on hyperbilirubinemic Gunn rats, UDCA in clinically relevant doses substantially increased turnover of unconjugated bilirubin, with a drop of bilirubin concentrations in the vascular bed comparable to that of phototherapy^32,33^; establishing a rationale for the use of UDCA in the treatment of severe neonatal jaundice. Indeed, this hypobilirubinemic effect of UDCA has recently been proven in several clinical studies on human neonates suffering from serious unconjugated hyperbilirubinemia, with no apparent adverse reactions attributable to administration of UDCA^34–38^.

It should be noted that concerns were raised related to the risk of development of necrotizing enterocolitis as the result of increased fecal BA concentrations^39^. On the other hand, human milk nutrition has been associated with a greater protective effect against infection-related events, including necrotizing enterocolitis^40^. Additionally, as seen from data on BA content in Tables 1, 2, ingested BA cannot in any case affect the fecal BA concentrations, since the contribution of BA from ingested breast milk to the total BA pool was only 0.002%.

Our cohort study as well as sporadic data, discussed above, consistently point to the safety of UDCA for breast-fed infants. Thus, our results support the recent recommendation for the use of UDCA during lactation, which was recently stated in Drugs and Lactation Database (LactMed) Ursodiol^4^ as well as in a recent Expert opinion paper by de Vries and Beuers^5^.

In conclusion, based on our detailed data as well as the other previous sporadic reports, UDCA treatment during lactation appears to be safe for breastfed babies. Hence, current therapeutic approach for treatment of cholestatic liver diseases in breastfeeding women should be reconsidered and treatment with UDCA should be included into the guidelines for the therapy of cholestatic diseases in breastfeeding mothers.

## Data Availability

The datasets used and/or analyzed during the current study available from the corresponding author on reasonable request.
