# Author Correction: Ursodeoxycholic acid use in lactating female patients is associated with clinically negligible concentrations of this bile acid in breast milk

**DOI:** 10.1038/s41598-022-26431-4

**Published:** 2022-12-16

**Authors:** Patrik Šimják, Tomáš Petr, Barbora Kaslová, Tomáš Fejfar, Petr Hůlek, Antonín Pařízek, Libor Vítek

**Affiliations:** 1 Department of Gynecology and Obstetrics, General University Hospital in Prague and 1st Faculty of Medicine, Prague, Czech Republic; 2 Institute of Medical Biochemistry and Laboratory Diagnostics, General University Hospital in Prague and 1st Faculty of Medicine, Prague, Czech Republic; 3 Department of Internal Medicine, University Hospital in Hradec Králové and Faculty of Medicine in Hradec Králové, Hradec Králové, Czech Republic; 4 4th Department of Internal Medicine, General University Hospital in Prague and 1st Faculty of Medicine, Charles University in Prague, U Nemocnice 2, Prague 2, 128 08 Czech Republic

Correction to: *Scientific Reports* 10.1038/s41598-022-24253-y, published online 15 November 2022

The original version of this Article contained an error in the Results,

“Taking into account the total BA pool in full-term newborn infants, which is approximately 60 mg^16^, a daily intake of 27 mg of UDCA would only represent 0.0005% of the total newborn BA pool.”

now reads:

“Taking into account the total BA pool in full-term newborn infants, which is approximately 60 mg^16^, a daily intake of 27 μg of UDCA would only represent 0.0005% of the total newborn BA pool.”

The original Article has been corrected.

